# Evolved but Not Fixed: A Life History Account of Gender Roles and Gender Inequality

**DOI:** 10.3389/fpsyg.2019.01709

**Published:** 2019-07-23

**Authors:** Nan Zhu, Lei Chang

**Affiliations:** Department of Psychology, University of Macau, Macau, China

**Keywords:** gender roles, life history strategies, parental investment, environmental unpredictability, competition

## Abstract

The rift between evolutionary psychology and the biosocial model of gender relations impedes a fuller understanding of gender roles and gender inequality. In a novel evolutionary account that complements both existing theories, we highlight life history strategies as intermediate mechanism linking distal environmental forces to variations in gender relations. Specifically, traditional versus modernized gender roles are seen as shaped by present-oriented versus future-oriented reproductive strategies, which are sensitive to uncontrollable morbidity-mortality risks. Gender inequality stems from a combination of present-oriented reproductive strategies adapted to high-risk environments and dominance hierarchies resulting from societal competition (i.e., the probability of obtaining resources desired by others through personal efforts). By contrast, gender egalitarian values develop as people increasingly enact future-oriented reproductive strategies in a competitive but orderly and controllable environment, which is conducive to prestige hierarchies. The current account provides novel interpretations of phenomena ranging from sex differences in mate preference, sociosexuality, and sexism to cross-cultural variability in marital systems and cultural practices. All of these serve to support the view that gender relations are evolved, changeable, and influenced by the interaction between ecological and social environments in ways predicted by the life history mechanism.

Both social constructionists (e.g., Wood and Eagly, 2012) and evolutionary psychologists (e.g., Buss and Schmitt, 2011) seek to elucidate two interrelated phenomena regarding gender relations: (1) the traditional sex-typed division of labor (“gender roles”), with women serving as homemakers and caretakers and men serving as providers and protectors (Shelton and John, 1996; Alesina et al., 2011), and (2) the power asymmetry between the sexes regarding the control of reproductive, economic, and political resources (“gender inequality”). Scholars from various theoretical perspectives have debated the distal origins and proximate causes of such gender roles and gender inequality, ranging from innate dispositions to historical construction and from sexual selection (Buss, 1995) to patriarchal social structures (Lerner, 1986; Hrdy, 1997). People’s take on such issues affects how they address existing gender inequality.

The current paper seeks to combine life history theory (Del Giudice et al., 2015) with sexual selection theory (Andersson, 1994). Our account maintains that gender relations are shaped by life history strategies that, on average, promote individuals’ present or future reproductive success in different ecological and social environments. When present-oriented reproductive goals are prioritized, sexual selection tends to exaggerate sex differences in the direction that favors traditional gender roles. Such a present-oriented strategy, when combined with dominance-based hierarchies shaped by agonistic, unrestricted competition (primarily among males), might contribute to gender inequality favoring men. In contrast, when future-oriented reproductive goals are prioritized, women and men are emancipated from the traditional “women as caregivers and men as providers” gender roles. A future-oriented strategy also facilitates the emergence of prestige-based hierarchies governed by non-agonistic, rule-regulated competition, which, in turn, reduce the power asymmetry between sexes. Therefore, gender relations are seen as largely malleable, rather than fixed, and can be systematically explained by examining the interplay between ecological and social environments in the human evolutionary history, cultures, ontogenetic environments, and transient situations. The current account does not justify or naturalize gender inequality by focusing on invariant sex differences, nor does it treat gender inequality as a purely sociohistorical invention. Rather, we aim at expanding existing evolutionary explanations and furthering the understanding of environment-contingent variations of gender relations.

## Previous Theories of Gender Relations

A number of explanations of gender roles and gender inequality have been proposed, ranging from the invention of the plow for intensive agriculture (Alesina et al., 2011) to the general progress of “modernization” (e.g., Inglehart and Baker, 2000). These individually highlighted elements of technology and historical process, however, are integrated in or explained by more comprehensive theories regarding the distal causes of gender roles and gender inequality, which can be broadly classified into evolutionary psychological accounts (e.g., Buss and Kenrick, 1998; Buss and Schmitt, 2011) and biosocial accounts (Wood and Eagly, 2002, 2012).

Evolutionary psychologists argue that at least some sex differences in human behaviors and psychological dispositions, especially those related to mate seeking and selection, are attributable to selective pressures of intersexual selection and intrasexual competition imposed by a number of adaptive challenges (Buss, 1995; Buss and Kenrick, 1998; Puts, 2010). These challenges include identifying reproductively valuable partners for both sexes, reducing paternity uncertainty for males, and eliciting partners’ parental investment in offspring for females (Buss and Schmitt, 1993; Buss, 1995). Males and females face different challenges due to males’ higher reproductive rate, which cause disparity in parental investment between sexes (Geary, 2000; Archer, 2009). Sexual selection’s “solutions” for these sex-specific challenges are seen as responsible for psychological dispositions relevant to mating and gender roles (Buss and Schmitt, 2011). For example, male-male competition for acquiring mates might lead to males’ propensity for aggression and risk-taking, while females’ selection of protective and high-investment males might allow them to be more dependent and risk-avoidant (Archer, 2009; Puts, 2010).

While different evolutionary psychology theories differ in their emphasis on various processes of sexual selection (e.g., male-male competition or intersexual selection), they all maintain that sex differences are ultimately produced by selection of inheritable traits, instead of non-genetic processes such as social learning (Buss and Schmitt, 2011). This is supported by strong consensus in behavioral genetics that almost all human psychological and behavioral traits show substantial genetic influence (Plomin et al., 2016). Moreover, males and females face differential sexual selection pressures due to sex-differentiated reproductive rates and costs (Trivers, 1972; Geary, 2002). For example, the heritability of sociosexuality (i.e., interest in casual sex) has been found to be higher among females than among males (0.43 vs. 0.26; Bailey et al., 2000), indicating that females’ greater sexual restrictedness is more influenced by genetic factors. This emphasis on genetic influences is frequently confused with genetic essentialism (i.e., regarding the superficial traits or social phenomena as determined by “genes,” which constitute fixed “essence” of organisms and social categories; Dar-Nimrod and Heine, 2011), which often generates misunderstanding of evolutionary psychology and evolutionary accounts of gender.

Contrary to this misguided impression, contemporary evolutionary psychologists actively reject genetic essentialism by acknowledging non-genetic, environmental inputs and phenotypic plasticity in human life history strategies (Geary, 2002). Despite this, early evolutionary psychological hypotheses linking invariant sexual selection processes directly to sex differences in mating fail to consider complex environmental effects, including gene-environment interactions (Bailey et al., 2000). It is also problematic to regard sex differences in mating as reflecting functionally distinct “modules” without considering the possibility that such sex differences in mating might be strategies adapted to different environmental challenges faced by each sex. More recent evolutionary accounts of the variations and sex differences in mating have taken into account factors such as operational sex ratio, pathogen pressure, resource availability, and cultural and legal contexts (e.g., Gangestad and Simpson, 2000; Schmitt, 2005; Lee and Zietsch, 2011). However, these separate environmental effects on mating are yet to be integrated in one theoretical framework and to be extended to account for gender relations (Buss and Schmitt, 2011). As a result, there is continued “essentialist” criticism lodged upon evolutionary psychological accounts of gender relations and worries that such accounts serve to legitimize gender inequality (Hrdy, 1997; Wood and Eagly, 2002).

As a competing account, Wood and Eagly (2002, 2012)’s biosocial model attributes gender roles and gender inequality to an interaction between “constraints and the opportunities imposed by each sex’s physical attributes and reproductive activities” (Wood and Eagly, 2002, p. 709) and social, technological, and economic factors. They reason that because sex-specific biological constraints render sex-typed division of labor more efficient than non-sex-typed collaboration, men become specialized in skilled activities that take them away from home while women focus on domestic tasks. Men achieve higher status *via* the monopoly of “warfare, agriculture, and production activities,” which generate far more material wealth than domestic labor (Wood and Eagly, 2002, p. 716). Eventually, the overgeneralization of the social reality of sex-typed division of labor to internal characteristics of women and men through “correspondent inference” cause people to construct and rationalize gender inequality (Wood and Eagly, 2012). The biosocial perspective also explicitly relates human mating preferences to gender inequality. However, unlike evolutionary psychological theories, the biosocial model regards sex-stereotyped mate preferences as resulting from socially constructed patriarchal systems, rather than sexual selection (Eagly and Wood, 1999).

Therefore, like the evolutionary accounts, the biosocial model acknowledges the existence of sex differences. However, like other social constructionist accounts (e.g., Hrdy, 1997), it resorts to a social constructionist explanation for gender relations and relevant psychological dispositions based on more recent sociohistorical factors, such as patriarchal systems adapting to sex differences in labor-participation efficiency (Wood and Eagly, 2002, 2012). However, plenty of ethnographic findings challenged this view, showing that many hunter-gatherer societies, for which biological constraints on female labor-participation efficiency is salient, exhibit relatively egalitarian gender relations (e.g., matrilineal tradition in a foraging and horticultural society on Vanatinai Island; Lepowsky, 1993; Agta women hunters; Goodman et al., 1985). Sex disparity in labor-participation efficiency also fail to explain the prevalence of gender roles in traditional societies that vary greatly in males’ contribution to subsistence (Marlowe, 2000), or the persistence of sexist gender roles in modern societies with minimal sex disparity in earning potentials (e.g., Evans and Diekman, 2009; Ebert et al., 2014). These limitations indicate that the biosocial model needs to be complemented by evolutionary mechanisms accounting for the possibility that women and men gain fitness to different degrees by adhering to unequal gender relations in some environments.

In summary, evolutionary psychology and the biosocial model share some common insights regarding gender relations while disagreeing upon the roles of sexual selection and sociohistorical factors. It is important to note that distal evolutionary mechanisms, such as parental investment (Trivers, 1972) and sexual selection (Andersson, 1994), are not mutually exclusive with more proximate and malleable biosocial and sociocultural factors. In complement to both theories, therefore, our life history account explicate the intermediate mechanisms governing the evolution and manifestation of gender relations in various ecological and social environments. This allow us to generate more specific hypotheses regarding individual and population differences in psychological attributes related to gender roles and gender inequality.

## A Life History Account of Gender Roles and Gender Inequality

Life history theory has been employed to explain human individual differences in a wide range of psychological and social traits based on tradeoffs between present and future reproductive success (Del Giudice et al., 2015). “Life history strategies,” which represent clusters of traits serving present- or future-oriented reproductive goals (including traits related to mating and gender roles), are sensitive to environmental risks throughout the life span, although early life experiences are particularly important (Chisholm, 1999). Thus, the life history perspective has more to do with explaining environment-contingent behavioral flexibilities (within the limits of reaction norms) than seeking specific evolutionary explanations for certain traits and behaviors observed in the modern environment.

Here, we partition “environmental influences” into two overarching forces: (1) extrinsic risks (often divided into harshness and unpredictability), which represent the morbidity and mortality threats that cannot be avoided through individual efforts (Ellis et al., 2009; Chang and Lu, 2017), and (2) societal competition, which represents the degree to which individual efforts affect a person’s access to resources desired by others in the same society. Both forces have been theorized as the fundamental shaping forces of inheritable, biological life history (MacArthur and Wilson, 1967) and are present throughout human evolutionary history. Accordingly, they shape acquired cultural values, which, in turn, lead to cross-cultural and regional variations in family relations, homicide trends, and moral perceptions (Hackman and Hruschka, 2013; Van Leeuwen et al., 2014). In modern times, extrinsic risks might take the forms of disasters, accidents, and disease, while societal competition usually takes the form of non-agonistic, prestige-based contests in educational and occupational arenas (Zhu et al., 2018), but may also manifest in agonistic, dominance-based contests (e.g., gangsters vying for higher ranks in mafia organizations, businessperson participating in price wars to seize market share). Early experiences of these events serve to calibrate individual life history strategies through development (Chisholm, 1999; Belsky et al., 2012). Meanwhile, these events might also serve as external cues to elicit behaviors congruent with the relevant life history strategy in transient situations (Griskevicius et al., 2011; Zhu et al., 2019).

### Effects of Extrinsic Risks on Gender Roles

In human society, harshness and unpredictability (e.g., famine, pathogens, disasters, and violence), through their indirect effects on families (e.g., parental harshness and insecure attachment), have been demonstrated to “accelerate” individuals’ life history, which manifests as earlier physiological maturation, earlier sexual debut, and earlier reproduction (Ellis and Essex, 2007; Belsky et al., 2010a,b, 2012; see Belsky, 2012 for a review). All of these factors effectively serve to prolong the female reproductive career and, ultimately, maximize the present reproductive success of both sexes. This thus increases the chance of an individual leaving at least one offspring before being hit by morbidity or mortality in dangerous environments (Ellis et al., 2009). As an exception to the purported link between extrinsic risks and accelerated life history, Schmitt (2005) found in a cross-cultural study that many indicators of extrinsic risks (e.g., high infant mortality rates) were negatively correlated with unrestricted sociosexuality at population level. However, it is important to note that sociosexuality (i.e., acceptance of casual sex) does not necessarily suggest present-oriented reproductive goals. In fact, most reasons provided by young adults for short-term sexual encounters seems unrelated to reproduction in affluent countries (Regan and Dreyer, 1999). This might explain why sociosexuality was negatively correlated with other indicators of present-oriented life history (e.g., teenage pregnancy rates) and positively correlated with gross domestic product and human development index (Schmitt, 2005).

However, the mentioned strategies impose long-term costs on an individual’s health, relationships, and offspring competitiveness (Chisholm, 1999; Geary, 2000, 2002). In particular, although both sexes share the benefits of a present-oriented reproductive strategy, women incur substantially greater costs of such a strategy. Reproductive activities such as pregnancy, breastfeeding, and childcare constitute a major obstacle to women‘s participation in most economic production activities (Wood and Eagly, 2012). Women’s relative vulnerability and helplessness during these critical periods also increase their reliance on men’s provisioning, even in traditional societies where women and men have, overall, similar contributions to subsistence (Marlowe, 2003). Moreover, because of the imbalance of initial parental investment in mammals (including humans) and the paternity uncertainty caused by concealed ovulation (Geary, 2000; Buss and Schmitt, 2011), mothers are predisposed to offer greater direct care to their offspring than fathers (Trivers, 1972). By contrast, men benefit more reproductively from seeking additional mates than they do from investing in existing offspring (Buss and Schmitt, 1993). Indeed, in most foraging societies, fathers provide much less direct care to their offspring than mothers do (Marlowe, 2000). This further widens the gap in parental investment between women and men such that the more offspring women have, the less time and energy they dispose of to spend on nonreproductive activities.

Notably, drastically imbalanced parental investment is not a fixed nature of humanity. Humans are exceptional among mammals in terms of paternal investment in offspring (Geary, 2000). However, shared parental care and extensive parenting (providing for and educating the children) have diminished returns in impoverished, dangerous environments (Quinlan, 2007), which might limit paternal investment, thus imposing greater burden of childcare on women in such environments. In other words, females’ higher reproductive costs would only contribute to imbalanced parental investment and traditional, sex-typed division of labor (i.e., women as caregivers and men as providers) when the costs of adhering to such rigid gender roles are deemed necessary to achieve higher reproductive success in the face of extrinsic risks.

### Effects of Societal Competition on Gender Roles

Since the ecological dominance of human species has overcome numerous extrinsic risks, including predators and cold climates, humans have become their own worst enemy because of societal competition (Alexander, 1989). The hyper-competitiveness of human society put selection pressure on the quality or competitiveness of offspring. This effectively heightens the reproductive costs for both sexes and favors delayed reproduction of fewer offspring, which allows excessive energy and resources to be allocated to growth, bodily maintenance, and parenting efforts (Geary, 2002; Del Giudice et al., 2015). The increase in childrearing costs can significantly affect human life history and parental investment because of the premature birth of human infants and prolonged dependency of human children (Walker et al., 2010). Moreover, with the accumulation and inheritance of wealth in stable and safe human societies, increased investment from both parents (especially fathers) not only improves offspring survival rates, but also enhances their skill development and social status (Mace, 1998; Geary, 2000). Thus, in a stable and safe environment, non-agonistic, prestige-based societal competition is conducive to an “arms race” of parental investment, as it becomes increasingly important for offspring to excel at skills (which help to earn fitness-enhancing prestige; Henrich and Gil-White, 2001). Meanwhile, women’s concealed ovulation, coupled with a pair-bonding mechanism, also motivate men to maintain long-term investment in their partners and offspring (Geary, 2000). Men who are more willing and capable to invest tend to out-reproduce those who are not in a competitive society, causing sexual selection to favor paternal investment (Geary, 2000). This would eventually narrow the parental investment gap between the sexes and foster “modernized,” flexible gender roles and gender equality.

It is important to distinguish societal competition from density-dependent competition. Early life history models regard competition as equivalent to population density or K-selection and opposite to extrinsic risks or r-selection, since populations facing high mortality risks generally have trouble maintaining high population density and, hence, intense competition (e.g., MacArthur and Wilson, 1967). Indeed, there is research demonstrating that people exposed to cues of population density (Sng et al., 2017) exhibited a more pronounced orientation toward long-term mating, later marriage age, lower fertility, and greater parental investment. However, these studies did not account for extrinsic risks. Moreover, the assumption that competition is inversely related to extrinsic risks does not hold for humans at the population level: many unstable, war-torn countries (e.g., Iraq, 93 people per km^2^) have higher population density than stable, prosperous countries (e.g., the United States, 36 people per km^2^; United Nations Department of Economic and Social Affairs, 2019). Given that higher reproductive rates induced by extrinsic risks might more than compensate for the population losses caused by extrinsic risks, harsh and unpredictable environments can also have high population density. Resource scarcity or uneven resource distribution in such environments, in turn, might intensify societal competition. Meanwhile, in stable and safe societies with a low disparity in wealth, high population density does not necessarily lead to high societal competition. Thus, a life history model of gender relations should take into account interactions between extrinsic risks and societal competition.

### Interaction Among Environmental Forces Affecting Gender Relations

Societal competition, when coupled with extrinsic risks, might not produce egalitarian gender relations for several reasons. First, offspring competitiveness gained from increased parental investment has diminished returns in the face of extrinsic threats (Quinlan, 2007). When facing frequent famines, disease outbreaks, or warfare, both sexes are likely to prioritize survival and present-oriented reproductive goals over future development, thus reinforcing traditional gender roles.

Secondly, such a combination of environmental forces would ratchet up male-male competition for present-oriented reproductive goals. Male-male competition often involves physical contests and even violence in the human evolutionary history (Archer, 2009; Puts, 2010). Indeed, early warfare between groups of men often involves the capture of women as slaves or “trophy” (Lerner, 1986). Reproductive competition among men has been one of the most fundamental incentives for fighting in pre-agricultural societies (Gat, 2000). Until the establishment of monogamy and anti-violence social institutions in larger-scale societies, the possibility of acquiring many wives through sheer force channeled societal competition toward a dominance contest favoring formidable and combative men. The resulting vicious circle of retaliation and revenges often leads to unpredictability of resource availability, which, in turn, reinforces agonistic competition even in resource-rich areas (Gat, 2000). This combination of extrinsic risks and dominance competition (not suppressed by anti-violence social institutions) reinforces the role of men as protectors and female’s dependence on male protectors. Meanwhile, in such environments, men also face increased threats of paternity uncertainty from same-sex competitors who pursue present-oriented reproductive goals *via* extra-pair mating. To maximize paternity certainty for their mating efforts, men might adopt controlling strategies to monopolize their female partners, ranging from violent coercion to “claustration, indoctrination, surveillance, gossip, inheritance rules, and laws” (Hrdy, 1997, p. 25). The resulting curtailment of female autonomy and mobility poses additional obstacles to women’s participation in production activities requiring the accumulation of skills and expertise through social exchanges (Wood and Eagly, 2002, 2012), which effectively cause women to be economically dependent on men.

The diminished return from parental investment and intensified male-male competition are not the only consequences of extrinsic risks, they also affect the expression of societal competition and the resulting social structure. The essential feature of power in human society is the ability to provide or withhold valued resources (Anderson and Berdahl, 2002), including access to mates. Societal competition enables successful individuals to control resources and/or mates without frequent challenges from subordinates, resulting in status hierarchies (Alexander, 1989; Cummins, 2006). There are two routes to high status in human societies. Individuals can earn others’ freely conferred deference (i.e., prestige) by exhibiting extraordinary skills or knowledge in valued domains and the willingness to share such information with conspecifics (Henrich and Gil-White, 2001). Such prestige-based societal competition depends on (1) the uniquely human cultural capacity and (2) future-oriented life history strategies to allocate more energy and resources to skill-building, knowledge-accumulation, and altruistic sharing. As a result, prestige competition and the resulting prestige hierarchy are fragile in the face of extrinsic risks, which would prompt individuals to pursue short-term goals of reproduction and mate monopoly, often through violence and domineering tactics (Daly and Wilson, 1990). In other words, humans are able to engage in societal competition on the prestige level and conform to prestige hierarchies in environments with low extrinsic risks to undermine the value of cultural transmission. However, they would resort to the similar kind of dominance competition observed in other species in harsh and unpredictable environments, which lead to dominance hierarchies enforced through violence and coercion (Henrich and Gil-White, 2001).

Dominance hierarchies shaped by agonistic forms of competition are more likely to favor men. Sex dimorphism in terms of physical strength, aggressiveness, and psychological competitiveness all favors men in combat or posing threats. Male-dominated power hierarchies, in turn, reinforce the traditional gender roles, which maximizes men’s reproductive success through the monopolization of (multiple or younger) female partners (Puts, 2010). Indeed, research has found that females show increased sexual attraction to males displaying dominant behavior (Sadalla et al., 1987). Ethnographic evidence also shows that polygynous mating systems are more prevalent in societies with a more uneven distribution of wealth and higher variations in male status compared with other societies (Marlowe, 2000). Data on reproductive success show that in contemporary traditional societies (hunter-gatherers and herder-gardeners) and ancient agricultural societies, men (but not women) show even larger variance and range of reproductive opportunities and mating success in more stratified societies (Betzig, 2012). In ancient agricultural societies, individuals with the highest status (e.g., emperors) usually maintain their status through dominance (Betzig, 2012). Thus, to the degree that dominance competition is prevalent in pre-modern societies, gender inequality should increase in these societies when they become more stratified. Moreover, when such dominance hierarchies are combined with a focus on present reproductive success in the face of extrinsic risks, women’s choices are constrained (Hrdy, 1997) in that their dependence on men might be the only recourse they have to promote their present-oriented reproductive goals. In polygynous societies that restrict women’s access to resources, women typically prefer to be one of the several co-wives of a prosperous man rather than the only wife of a poor one (Betzig, 1986).

Societal competition expressed as non-agonistic contests based on skills and altruism can also benefit high-prestige individuals reproductively (Henrich and Gil-White, 2001). One research showed that women preferred high prestige, low dominance men in long-term relationships, but a high dominance men was preferred in short-term relationships (Snyder et al., 2008). This seems to apply to traditional societies as well. Research on Amerindian societies, for example, revealed that men’s prestige, but not dominance, indirectly predicts their number of offspring through their female partners’ age at first reproduction (von Rueden et al., 2011). Similarly, a more recent study on BaYaka Pygmy hunter-gatherers showed that prestige (indicated by popularity in a gift game) positively predicted men’s success in the mating market (Chaudhary et al., 2015). More importantly, to the degree that prestige competition requires social skills more than physical prowess, men are not at advantage in such competition, since women generally score higher than men on altruism, agreeableness, and social skills (MacDonald, 1995; Petrides and Furnham, 2000). When combined with socially-imposed monogamy, contraceptive technologies, and increased economic niches that depend less on physical strength, prestige competition is likely to transform traditional sex-typed social roles into sex-flexible ones and foster a gender egalitarian social structure.

The above analyses lead us to a series of predictions regarding gender roles and gender inequality (see Table 1 for a summary of theoretical predictions in environments varying in these two dimensions). Specifically, in societies that are dangerous and unstable but without intense competition, a traditional sex-typed division of labor would be prevalent. However, women in such societies would enjoy a similar social status as men. By contrast, societies that are safe, stable, and competitive would foster modernized gender roles and gender egalitarian values. When extrinsic risks are combined with societal competition, however, present-oriented reproductive goals would be prioritized, contributing to traditional gender roles. In addition, male-male competition in harsh and unpredictable environments would promote male monopoly over resources and dominance-based social hierarchies that favors male, ultimately perpetuating gender inequality. Finally, in societies that are stable and safe, but non-competitive, men would attempt to realize their reproductive potential with present-oriented reproductive goals, while women would prefer lower reproductive costs with future-oriented reproductive goals. A compromise would probably result in moderate gender role segregation. Meanwhile, the absent of male-dominated social hierarchy would allow some degree of gender equality.

**Table 1:** Summary of theoretical predictions of reproductive strategies, social structures, and gender relation outcomes in various environmental conditions.

	Low societal competition	High societal competition
Low extrinsic risks	Present-oriented reproductive strategies Low social stratification Mixed gender roles Low gender inequality	Future-oriented reproductive strategies Prestige-based social stratification Modernized gender roles Low gender inequality
High extrinsic risks	Present-oriented reproductive strategies Low social stratification Traditional gender roles Low gender inequality	Present-oriented reproductive strategies Dominance-based social stratification Traditional gender roles High gender inequality

## Implications and Predictions of the Life History Account

Two general implications can be deduced from the preceding analysis. The first and most crucial one is that the social and behavioral biases that result in gender roles and gender inequality are evolved but not fixed. Rapid changes in gender relations can occur due to cultural evolution (Newson and Richerson, 2009) and more nuanced environmental changes within a society. Notably, this might explain numerous findings regarding sex differences in mate preferences (e.g., Buss et al., 2001; Chang et al., 2011), sociosexuality (Schmitt, 2005; Kandrik et al., 2015), and sexism (Glick et al., 2000; Glick and Fiske, 2001). Second, the interaction between extrinsic risks and societal competition underlies parts of the variations in gender roles and gender inequality. This enables us to interpret in novel ways historical and cross-cultural variations in marital systems, parental investment, and cultural practices (e.g., foot-binding practice and corset fashion).

### Gender Roles Are Evolved and Changeable

Sex differences in mate preferences might elucidate the prevailing gender roles in society. Specifically, male preferences for women’s domestic skills and fertility reflect traditional female gender roles as homemakers and caregivers. This complements women’s preference for men’s social status and provisioning abilities, which reflects traditional male gender roles as providers and protectors. Previous research did demonstrate such sex differences in mate selection standards (Buss, 1989, 1995; Buss and Schmitt, 1993). In general, women have been reported to prioritize financial prospects and social status, whereas men have been revealed to prioritize youthfulness and physical appearance (Shackelford et al., 2005; Furnham, 2009). This pattern persisted in long-term mate selection efforts among wider ranges of potential mates and in “budgeted” mate selection tasks (Li et al., 2002, 2011), prompting Li et al. (2002) to regard such preferences as universal “necessities.”

These well-documented mate preferences are considered as strategies derived from sex-specific adaptations to sexual selection pressures (Buss and Schmitt, 1993). However, this does not mean that the magnitude of sex differences in mate preferences is necessarily universal or fixed. In fact, several cross-sectional studies tracing mate preferences in major economies over the past few decades have shown steady decreases in sex differences (in United States 1939–1996: Buss et al., 2001; China 1980s–2008: Chang et al., 2011; Brazil 1984–2014: Souza et al., 2016). In all these studies, financial prospect was increasingly valued by both sexes, particularly men (which might reflect increasing societal competition), whereas men attached lower importance to domestic skills and virginity. This, to some extent, reflects the prevalence of future-oriented life histories and a gradual modernization of gender roles in these societies, which coincides with long periods of peaceful and stable economic growth after World War II in increasingly competitive societies.

In addition, mate preferences also vary across societies and appear to be contingent on extrinsic risks (e.g., pathogens, resource scarcity, warfare). Research has shown that in such dangerous environments, women prefer men with indicators of good genes (e.g., symmetrical features; Gangestad and Simpson, 2000) or dominance status (Cummins, 2006), in order to increase the survivability of their offspring. For example, women in Tanzania’s Hadza hunter-gatherer groups exhibited increased preferences for symmetry in opposite-sex faces (especially when they were pregnant or nursing) compared with people in the United Kingdom (Little et al., 2007). Similarly, in a 29-country cross-cultural study, pathogen prevalence was associated with greater perceived importance of attractiveness for both sexes and lower perceived importance of paternal investment for women (Gangestad and Buss, 1993). In a more recent study, women’s preferences for male facial masculinity were negatively correlated with the national health index (DeBruine et al., 2010). These findings challenged an over-simplified view of sexual selection that overlooks environment-induced variations in the sex differences in mate preferences, which help to shape gender roles in different societies.

Even within a society, individuals’ mate preferences differ in predictable ways. Studies have shown that women’s financial independence and power (Moore et al., 2006) and educational attainment (Kasser and Sharma, 1999) were negatively related to the importance they attached to financial prospects in their mate preference. Similarly, Lu et al. (2015) observed that women with high socioeconomic status or living in urban areas, compared with those with low socioeconomic status or living in rural areas, prioritized good father attributes (e.g., caring, loves children) over good provider (e.g., successful career, ambitious) or good gene attributes (e.g., masculine, athletic). Finally, experimental studies found that men identified as present-oriented in life history strategy expressed greater preference for fertility and good-gene-related mate qualities, and were more sensitive to neoteny female faces representing fertility. Overall, the above evidence is compatible with the life history account of gender roles, indicating that present-oriented reproductive strategies might contribute to traditional “vulnerable females and protecting males” gender roles in mating. Conversely, future-oriented reproductive strategies supported by gender-equal competition foster modernized mate preferences and gender roles.

As another aspect of human mating, sociosexual orientation reflects individuals’ acceptance of uncommitted sex (Simpson and Gangestad, 1991). As discussed earlier, population-level sociosexuality aggregating between sexes may not reflect present-oriented reproductive goals or gender relations (e.g., in gender egalitarian societies, women tend to be more sexually unrestricted whereas men show the opposite trend; Schmitt, 2005). Nevertheless, from the life history perspective, we predict sex differences in sociosexuality should be a function of the prevalence of present-oriented reproductive goals. In support of this prediction, Schmitt (2005) found that the magnitude of sex differences in sociosexuality did differ across countries. Specifically, teenage pregnancy rate and fertility rate, both reflecting present-oriented reproductive goals, were both negatively correlated with women’s sociosexuality but not with men’s sociosexuality, contributing to larger sex differences in sociosexuality. Similarly, a more recent survey found that in the United States, women, but not men, reported lower desire for uncommitted sex in states with more demanding environments (e.g., higher teenage pregnancy rate, lower life expectancy), although the same sex difference was not found for sociosexual attitudes or behavior (Kandrik et al., 2015).

Finally, the life history account also offer insights into sex differences in sexism, which is defined as hostile or “benevolent” judgments of the opposite sex that justify treating people according to their sex (Glick and Fiske, 2001). Sexism is often regarded as a justification of traditional gender roles and the patriarchal system (Barreto and Ellemers, 2010). However, it also reflects psychological adaptations of both sexes to advance their reproductive interests in the face of extrinsic risks and societal competition. Previous studies generally revealed that men score higher than women on both hostile and benevolent sexism (e.g., Glick and Fiske, 2001). This is understandable from the evolutionary perspective, as men benefit more from justifying traditional gender roles that facilitate present-oriented reproductive goals because of their higher reproductive rates. Accordingly, men should be more “motivated” to show more pronounced sexist attitudes than women do, especially when facing increased extrinsic risks. In addition, in societies with greater between-sex conflicts over parental investment (which reflects present-oriented reproductive goals), men should exhibit higher hostile sexism whereas women should reject such hostile sexism. Consistent with this prediction, Glick et al. (2000) found that, across 19 nations, men’s hostile sexism is associated with increased sex gap in acceptance of sexism. Moreover, using the World Values Survey data, Newson and Richerson (2009) showed that countries with earlier decline in fertility (reflecting cultural endorsement of future-oriented reproductive goals) exhibited higher gender empowerment attitudes (opposite to sexist attitudes) than those with later decline in fertility. These findings, although preliminary, suggest life history strategies might affect attitudes and beliefs about gender relations.

In summary, multiple lines of evidence suggest that sex-differentiated mate preferences, which support traditional gender roles, likely represent present-oriented reproductive strategies adapted to extrinsic risks. Similarly, sex differences in sociosexuality and sexism are also better conceived as evolutionary products of flexible life history strategies than fixed aspects of human nature or purely sociohistorical artifacts. However, further research is needed to support detailed hypotheses.

### Interaction Between Unpredictability and Societal Competition

The current life history account goes beyond acknowledging environmental influences on gender relations through life history strategies. We also seek to predict nuanced patterns of cross-society and within-society variations in gender relations by examining distal environmental effects, which also operate in the human environment of evolutionary adaptedness (EEA). In particular, the interaction between extrinsic risks and societal competition might shed light on an array of cultural phenomena (mostly documented in ethnographic studies) relevant to gender roles and gender inequality.

Small-scale, nonagricultural societies, some of which probably resemble those inhabiting the human EEA (Volk and Atkinson, 2013), generally face high extrinsic risks. Two primary sources of uncontrollable, extrinsic risks in these societies are infant/child mortality and violence (e.g., blood revenge, raids, and full-scale warfare), which are found to be more prevalent in traditional societies than in modern societies (Chagnon, 1988; Kramer and Greaves, 2010). A study on contemporary hunter–gatherers and historical data estimated that the infant mortality and child mortality rates in the human EEA are 27 and 47.5%, respectively (Volk and Atkinson, 2013). Meanwhile, on the basis of his study on Yanomami tribal societies, Chagnon (1988) reported that deaths caused by violence accounted for approximately 30% of adult male mortality and that nearly 70% of adults aged 40 years or older had lost at least one close relative to violence. Higher reproductive efforts to offset elevated juvenile and adult mortality rates might lead to present-oriented life history strategies and more imbalanced parental investment between the sexes, which would contribute to the perpetuation of traditional gender roles in nonagricultural societies. Consistent with this prediction, in 77% of pre-industrial societies analyzed by Kelly (1995), men contributed more than women did to subsistence, which is consistent with the traditional male role as primary providers.

Nevertheless, the proportion of polygynous mating systems, which is usually associated with constraints on women’s autonomy (Hrdy, 1997), differs greatly among these societies. Among hunter-gatherer societies, which had the lowest population density and social stratification, only 21% were classified as generally polygynous, compared with 41% among pastoralist societies, 39% among horticulturalist societies, and 25% among agricultural societies (Marlowe, 2000; see Apostolou, 2007 for a similar estimation of the rate of polygyny across 190 hunter-gatherer societies). Among nonagricultural societies (including hunter-gatherer, horticulturalist, and pastoralist societies), the degree of polygyny was positively linked to social stratification (Marlowe, 2000; Betzig, 2012), which reflects the variations of male social status. Given people in these societies also face high degree of extrinsic risks, intense societal competition is more likely leads to violent conflicts and dominance hierarchies, rather than skill contests and prestige hierarchies. In other words, in societies facing high extrinsic risks, less intense societal competition (shown as lower levels of social stratification) might actually prevent the emergence of extreme power asymmetry favoring men. This seemed to be the case for most hunter-gatherer societies, whose subsistence style cannot support a dense population necessary for more complex social structures (compared with horticulturalists and pastoralists, hunter-gatherers are the lowest in social stratification; Marlowe, 2000), and the degree of polygyny is typically low (e.g., Marlowe, 2000, 2004; Apostolou, 2007). Additionally, the hunter-gatherer practice of equitable sharing of large games among households without favoring hunters’ families (Hawkes et al., 2001) might also prevent uneven distribution of wealth and the emergence of any form of hierarchy. Therefore, consistent with our theoretical prediction, an absence of dominance hierarchies might explain why some hunter–gatherers are less susceptible to gender inequality in marital system, even though they do adopt traditional gender roles (Marlowe, 2000, 2004).

Polygyny is also rare in agricultural societies, but this might be due to socially imposed monogamy, rather than indicating equal power between sexes in such societies (Alesina et al., 2011). In societies that practice intensive agricultural labor, women usually have a far lower status than do men (Alesina et al., 2011). Ethnographic research demonstrated that agricultural societies had a lower degree of female affairs than did any type of nonagricultural societies (Marlowe, 2000). This indirectly reflects effective male control of women’s reproductive activities. Furthermore, some cultural practices in historically agricultural cultures appear to exaggerate women’s vulnerability, powerlessness, and need for protection while restraining their mobility. This includes the foot-binding practice in feudal China (Carroll, 2009) and the fashion of corset and tightlacing in 19th century Europe (Steele, 1999). Both practices are sexually appealing to men but concurrently limit female mobility: for example, foot-binding causes difficulty in walking among women without the support of their shoes (Bossen, 2004). A common feature of these two cultural practices is that they emerge in highly stratified societies with male-dominated hierarchies before the demographic transition (Lee, 2003). This is compatible with our postulation that a combination of intensive male-male competition for dominance status and high reproductive efforts contributes to gender inequality.

In contrast, these practices rapidly declined and gender egalitarian values replaced traditional values that suppress women’s freedom as Europe and East Asia went through industrialization and demographic transition. Two interrelated reasons might account for this cultural practice and cultural value transition. First, increased concentration of population in urban areas and in industrial jobs leads to large-scale cooperative societies comprising mostly strangers, instead of kin-groups (Henrich et al., 2010). From the perspective of cultural evolution, cultural transmission from relatives (which usually encourages present-oriented reproductive goals) declined in such modern environments, allowing future-oriented reproductive goals to prevail in these competitive societies (Newson and Richerson, 2009). This may constitute a precondition for women’s (and men’s) emancipation from traditional gender roles. Meanwhile, third-party punishment and policing against violence are vital for the stability and order in such large-scale societies (Henrich et al., 2010), which restrain dominance-based competition and promote prestige-based competition. Together with future-oriented reproductive strategy, this shift toward prestige competition might render male-dominance cultural practices and relevant gender inequality values obsolete. Overall, these analyses show that cultural practices and values related to gender relations are not merely arbitrary, sociohistorical constructions. Rather, they might embody life history strategies and cultural adaptations that are sensitive to extrinsic risks and societal competition.

## Conclusion

Our life history account complement existing theories about gender relations by: (1) emphasizing the fact that the evolutionary processes, including sexual selection, that shape traditional gender roles and gender inequality are flexible rather than fixed, and (2) providing specific predictions regarding how these processes are contingent on the interaction between extrinsic risks and societal competition. Future research is needed to improve the evidentiary status of environmental influences on gender relations, and it faces several challenges.

First, identifying the sources of extrinsic risks in modern environments while ruling out confounding genetic effects can be difficult. Previous research has examined familial resource insecurity (e.g., income-to-needs ratio; Belsky et al., 2012), life changes or negative life events (e.g., Brumbach et al., 2009; Zhu et al., 2018), parental absence (e.g., Chang and Lu, 2018), and self-reported exposure to violence (e.g., Brumbach et al., 2009). None of these measures reflects pure environmental influences, though, as behavioral genetics studies has shown that environmental risks in shared family environment (e.g., parenting styles, maternal attachment) are partially explained by genetic effects (Plomin et al., 2016). However, extrinsic risks assessed in the form of uncontrollable life events (e.g., death of a spouse) are less likely to confound with genetic influences than controllable life events (e.g., financial problems; Plomin et al., 2016). Thus, to test our aforementioned hypotheses, it is important to use assessments with smaller genetic variance or heritability, and to interpret the results with caution when such assessments likely involve controllable aspects of environment.

Secondly, further theoretical and empirical works are needed to elaborate different evolutionary pressures for and different social developmental consequences of dominance versus prestige competition in a life history framework. As two forms of societal competition or status-seeking strategies, dominance and prestige are conceptually separable (Henrich and Gil-White, 2001). However, it is entirely possible that any status hierarchy conveys both dominance status and prestige status to various degrees, and they may lead to or end up being mixed with each other in most cases (Henrich and Gil-White, 2001). Dominance and prestige as different means to status are also not tied to certain type of societies or subsistence style. On the one hand, traditional societies are not all structured as dominance hierarchies derived from belligerent competition. Prestige-based competition can be an important way to achieve greater reproductive success without wealth accumulation in some hunter-gatherer societies (e.g., Chaudhary et al., 2015). In other hunter-gatherer groups, such as the Hadza in northern Tanzania, there is no clear dominance or prestige hierarchies (Marlowe, 2004), although the altruistic sharing of meat by good hunters can be seen as instances of prestige-based competition (Hawkes et al., 2001). In these groups, monogamy is the norm (with fairly high divorce rate) and women typically have a say in important decisions (indicating some level of gender equality; Marlowe, 2004). In industrialized societies, on the other hand, although dominance hierarchies are largely suppressed, dominance competition still exists in some areas and continues to affect at least short-term mating preferences (e.g., Snyder et al., 2008).

We propose that the relative importance of dominance and prestige in societal competition might have more to do with life history tradeoffs in the face of extrinsic risks. Specifically, dominance competition might prevail in high-risk environments, as present-oriented reproductive goals prompt male–male competition and agonistic confrontations over resources (Gat, 2000). Prestige competition, by contrast, might be more prevalent when extrinsic risks are low, as skill development and altruism both require future-oriented somatic efforts in relatively stable environments. Individual differences in dominance-based or prestige-based status-seeking strategies might also depend on individual life history strategies (accelerated life history might prompt individuals to rely more on dominance). In this way, life history strategies manifesting at the culture level might affect the nature of status hierarchies, which, in turn, influences gender relations. As a caveat, since prestige competition is largely related to humans’ cultural capacity (Henrich and Gil-White, 2001), there is no good parallel in non-human species. Thus, more theoretical and empirical works are needed to extend the life history framework to the potential tradeoff between dominance and prestige in status dynamics and social structures.

A third challenge lies in recognizing individual differences in susceptibility to environmental influences at different levels (Belsky, 2012). For instance, experimental evidence shows that situational cues of extrinsic risks might induce more present-oriented reproductive planning in individuals with childhood or chronic exposure to resource insecurity than in those who did not experience resource insecurity (Griskevicius et al., 2011). Similarly, a recent study revealed that individuals facing chronic resource disadvantages reduced their prosocial behaviors when exposed to competitive scenarios, whereas the opposite was true for advantaged individuals (Zhu et al., 2019). Moreover, although the current account focus on only two overarching environmental forces, it does not rule out other environmental factors with more proximate influences on gender relations, such as socially-imposed marital systems, the availability of contraception and alloparents, cooperative breeding, and advances in education, legislation, and technology. These factors might affect individuals’ exposure to extrinsic risks and societal competition, and lead to social structural variations that affect behaviors and psychologies underlying gender relations. These more proximate factors complement trait plasticity shaped by life history trade-offs influenced by chronic experiences of extrinsic risks and societal competition. Taking these into consideration provides additional directions for future research on individual-level and society-level variations in gender relations.

A fourth challenge is to distinguish gender inequality from gender roles—although they are occasionally intricately related to each other (see Eagly and Wood, 1999)—and to avoid the pitfall of taking all gender roles as embodying gender inequality. As evidenced by ethnographic studies, gendered division of labor (e.g., male provisioning) and practices of gender inequality (e.g., polygynous mating systems) might stem from independent environmental pressures (Marlowe, 2000). This also cautions against the assessment of gender inequality by using a single indicator, because gender inequality might take various forms and even be concealed in ostensibly benevolent social arrangements.

Finally, our position should not be mistaken as yet another version of gender essentialism. We concur with the biosocial model (Wood and Eagly, 2002, 2012) and other social constructionist accounts (e.g., Lerner, 1986) in that gender inequality should not be justified simply because it has evolutionary roots. Nor do we simply regard sexist gender roles and gender inequality as sociohistorical artifacts that are bound to be eliminated by “social progress” or “modernization.” Events such as violent revolutions, wars, and internal conflicts might disrupt the social progress toward gender equality. Moreover, even peaceful, modern societies are not free from extrinsic risks in the forms of crimes, family discords, and social commotions, which might bias societal competition toward masculine dominance. This might explain the unyielding prevalence of sexist gender roles and gender inequality in postindustrial countries that have long advocated gender egalitarian ideologies. To make people truly want gender equality and flexible gender roles, the current life history account suggests that we should begin by transforming our society toward a stable and safe one with non-agonistic, prestige competition.

## Author Contributions

NZ and LC conceptualized the manuscript. NZ prepared the first draft and LC revised and finalized the manuscript.

### Conflict of Interest Statement

The authors declare that the research was conducted in the absence of any commercial or financial relationships that could be construed as a potential conflict of interest.
